# Understanding Osmolality as a Critical Process Parameter in Mammalian Muscle Cell Culture and Biomanufacturing

**DOI:** 10.1021/acsomega.6c00350

**Published:** 2026-07-18

**Authors:** Mirinal K. Rayappa

**Affiliations:** James Watt School of Engineering, 3526 University of Glasgow, Glasgow, Scotland G12 8QQ, U.K.

## Abstract

Osmolality is a critical yet frequently underrecognized physicochemical parameter in mammalian cell culture systems and remains insufficiently treated as a formal process control variable in bioprocess engineering. To establish its importance comprehensively, this article opens with the physicochemical basis of osmolality and the nonidealities of its calculation and measurement in complex media (including method-dependent differences between freezing point depression and vapor pressure depression), arguing that measurement fidelity is central to its interpretation. It then synthesizes evidence supporting the importance of culture medium osmolality, specifically in mammalian muscle cells and tissues. It emphasizes their narrow operating window and the effects of osmotic excursions within that window on their proliferation, differentiation, and fusion. Building on this biology, the article introduces media composition and “chemical space” as an engineering framework for osmolality control, showing how salts, buffers, amino acids, and macromolecular additives consume a “finite osmotic budget” within this chemical space, thereby constraining serum-free, cost-optimized myogenic formulations. The narrative then moves to the physical, chemical, and material drivers of common osmolality drift, such as CO_2_/bicarbonate–pH coupling, temperature-linked component instability, evaporation in small-volume platforms, and scaffold/bioink interactions that can create bulk or local osmotic microenvironmental niches ranging from plates to bioreactors. Finally, this article argues for the use of time-resolved osmolality measurement as a bioprocess state indicator during scale-up, to support more robust process control. Throughout this work, examples from muscle lineage studies and broader mammalian bioprocessing studies are used to derive transferable principles applicable to the growing field of cultured meat and the general cell/tissue culture practices.

## Graphical abstract

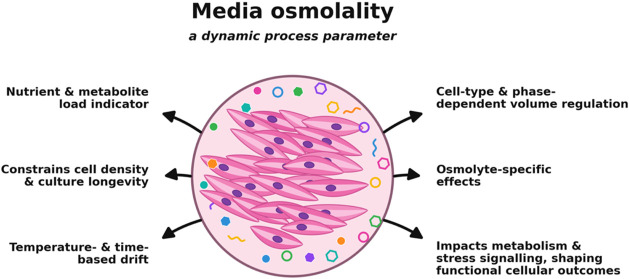

## 1 Introduction

Osmolality is a fundamental physicochemical variable in mammalian cell culture, governing water activity, solvent flux, and cell volume regulation.^1,2^ These processes directly influence proliferation kinetics, intracellular energy balance, metabolic fluxes, differentiation efficiency, and ultimately the productivity and robustness of cell-based bioprocesses.^3,4^ Despite this central role, osmolality remains markedly underreported in the muscle cell culture literature (and the wider mammalian cell studies), including within the rapidly expanding field of cultured meat. While pH, temperature, and dissolved oxygen are routinely tracked, osmolality is often treated as a static formulation property rather than a dynamic process parameter requiring deliberate cell-specific monitoring and control.

The inconsistent reporting of osmolality is particularly concerning for muscle-lineage cells (e.g., C2C12, primary myoblasts, bovine satellite cells), which often operate within narrower functional osmotic windows than many commonly used biomanufacturing cell lines (Table 1). Their (1) lack of a protective cell wall (instead, they have a membrane plus connective tissue sheaths (endomysium, perimysium, epimysium)), (2) reliance on tuned ionic gradients (their function depends on excitation–contraction coupling, which requires precisely regulated ionic fluxes (Na^+^, K^+^, Ca^2+^)), and (3) dependence on cytoskeletal integrity for fusion and contractile maturation (muscle function and maturation depend on the integrity of the cytoskeletal architecture, which enables fiber alignment) collectively heighten their sensitivity to osmotic perturbation.^5,6^ Even though C2C12 is frequently viewed as a “robust workhorse” in routine lab culture practices, this apparent resilience is often conditional on high-serum growth media, serum-based differentiation regimens, and frequent medium changes, which can obscure actual osmolality-induced physicochemical drift.^15^ Moreover, muscle cells can remain viable, while myogenesis can be functionally perturbed, i.e., with osmolality responses being cell type/state-dependent, they engage in various osmotic stress programs (e.g., NFAT5-linked responses), adaptability programs that can potentially, in the case of myogenic cells, compromise migration and differentiation-relevant pathways.^7^ This is directly relevant to bioprocess applications such as cultured meat, where primary livestock satellite cells cultured in serum-free or chemically defined media (combined with inherent donor and species-specific cell variability and the loss of serum-derived formulation properties) operate within relatively tighter process margins and are more susceptible to osmotic drift.^8,9^ Therefore, robustness should not be inferred from the C2C12 myoblast’s behavior alone; instead, osmolality tolerance and cell phase-specific set points should be empirically mapped for the actual bioprocess-relevant cell sources even under serum-free and scale-up conditions.

**Table 1:**
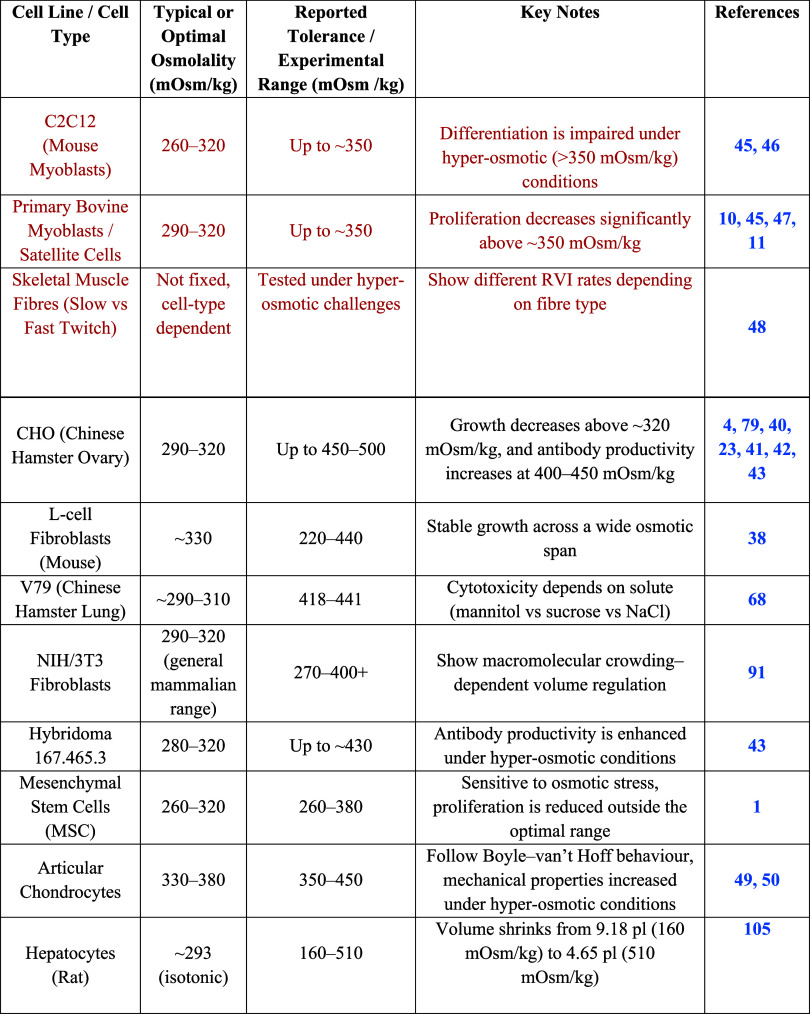
Reported Osmolality Ranges for Mammalian Cell Lines Relevant to Bioprocessing^a^

a Red color: muscle cell lines/types. Black color: other mammalian cell lines/types.

Most recent primary studies and reviews on serum-free or chemically defined media for bovine satellite cells including the widely used formulations such as Beefy-9 and other serum-free/defined media tend to report detailed media composition (e.g., growth factor substitutions) alongside performance readouts such as sustained expansion across passages, doubling time, differentiation outcomes etc.,^10–12^ yet measured osmolality is not consistently reported as an experimental variable in the final working formulation. Similar gaps arise in studies examining extracellular matrix interactions,^13^ scaffold/coating choices and scaled culture formats (e.g., microcarriers),^14^ or differentiation protocols for myogenic cells, where proliferation, cell cycle behavior, lineage markers, and fusion indices are quantified without establishing whether osmotic stress contributed to the observed phenotypes.

Even outside the cultured meat context, standard C2C12 culture protocols, vendor manuals, and widely circulated laboratory method papers commonly specify basal medium identity and serum percentages for growth and differentiation (e.g., DMEM + %FBS for expansion, DMEM + %horse serum for differentiation), and yet the measured osmolality of the final, supplemented medium is rarely reported before and after culture, nor correlated with cell phenotype (e.g., serum withdrawal, differentiation induction, glucose supplementation) that can materially shift the media osmotic load.^15–17^ This omission contrasts with the historical foundations of mammalian cell culture, where balanced salt solutions (e.g., Ringer’s solution)^18^ emphasized matching osmolarity to cellular physiological conditions (∼290 ± 30 mOsm/kg) to maintain cell viability.^3,19,20^ Beyond the ingredient-dense and serum-free/optimized formulations having dynamic solute interactions, osmolality is also inherently shaped by many factors such as evaporation, CO_2_/bicarbonate equilibria, metabolite accumulation (e.g., lactate, ammonia), solute release from scaffolded systems, or processing/sterilization-related leachables from biomaterials (explained in later sections),^21–24^ thus making it an important but often untracked covariate that can confound the interpretation of cellular growth, metabolism, and differentiation outcomes. Because these drift mechanisms depend strongly on culture format, the magnitude and direction of change can vary across common culture platforms and time points, such as the following:

- **Multiwell/plate and dish culture (including microdrops under oil)**: Even in oil-covered microdrops and in dry benchtop incubators, evaporation increases osmolality within a few days, and factors such as humidity and dish or oil volume strongly influence the extent of this drift. Small volumes (20–60 μL) are especially susceptible, with osmolality typically rising from ∼275–280 to ≥300 mOsm/kg within 2–6 days, a change that has been shown to affect developmental outcomes in sensitive systems.^25–28^

- **Flasks/plate culture under ambient CO**_**2**_: CO_2_ dissolution and bicarbonate buffer chemistry can contribute to osmolality drift even when pH appears controlled. This coupling is an underrecognized source of variability and is treated mechanistically, with its quantitative implications for osmolality and pH explained further in later sections.^29–31^

- **Bioreactors (batch, fed-batch, perfusion)**: At a large scale, rising pCO_2_ and accumulating feeds/byproducts can co-occur with osmolality drift, accompanied by reduced growth performance, reinforcing the need for cell phase-specific medium osmolality measurement and reporting.^32^ In this context, batch cultures operate without medium exchange after cell inoculation; fed-batch processes incorporate discrete or continuous nutrient feeds without cell removal; and perfusion systems enable continuous medium exchange with cell retention, allowing tighter control of the extracellular environment.

In high-density or long-duration cultures, which are common for cultivated meat workflows, osmolality can drift by tens of mOsm/kg (e.g., CHO cell culture data show osmolality increases of ∼27–51 mOsm/kg at elevated pCO_2_ with pH/osmolality compensation and larger increases when not compensated), and similar order shifts can arise in small-scale systems through evaporation-driven concentration of solutes during extended culture.^23,40^ Without time series measurements or phase-specific monitoring (e.g., late proliferation, differentiation induction, postfusion), it becomes difficult to attribute observed phenotypes to the corresponding nutrient availability, growth factor supplementation, or underlying osmotic drift. Hyper-osmolality drives rapid cell shrinkage and intracellular macromolecular crowding, shifting cellular metabolism toward a catabolic, energy-stress state (e.g., reduced ATP/energy charge, increased phosphocreatine breakdown, and elevated glycolytic byproducts such as lactate). Such responses unfold on rapid time scales of seconds to minutes, consistent with the intrinsic metabolic responsiveness of mammalian cells where reported oxygen utilization rates across cultured mammalian cells span a broad range from <1 to >350 amol cell^–1^ s^–1^, reflecting substantial heterogeneity across cell types and physiological states (for example, C2C12 mouse myoblasts display oxygen consumption rates of ∼3.7 amol cell^–1^ s^–1^).^105^ Oxygen utilization rates are cited here not as a direct measure of osmotic stress by any means but to provide a physiological context for the rapid metabolic responsiveness and energetic capacity that, in this case, can constrain cellular adaptation to hyperosmotic perturbations, including important metabolic consequences arising due to cell size and volume changes occurring on ∼30 s (in rat hepatocytes) time scales in a cell type-dependent manner.^105^ In contrast, hypo-osmolality promotes cell swelling and can bias cells toward a more anabolic state. When hyperosmotic stress is severe or sustained, it can trigger oxidative stress and subsequent damage responses (e.g., DNA and protein damage, mitochondrial depolarization), leading to cell cycle delay or arrest and ultimately apoptosis.^33^ Overall, departures from the tolerable osmotic window reprogram metabolism and stress signaling, thereby impacting growth performance, function, and viability.

For these reasons, this article frames osmolality as a critical process parameter in myogenic culture, rather than merely a fixed property of a media recipe. It proceeds from explaining the physicochemical basis and measurement/calculation nonidealities of media formulations to muscle cell and tissue responses across proliferation, differentiation, and fusion, to “chemical space” as a framework for media design, and finally to the main drivers of osmolality drift from CO_2_/pH coupling and temperature effects, metabolites, and scaffold/material interactions across plates to bioreactors, with time-resolved osmolality proposed as a practical scale-up state indicator. Throughout, each concept is supported by case studies, drawing examples from muscle cell systems wherever available and extending the logic with well-characterized mammalian bioprocess literature when muscle-specific data are sparse, so that the resulting framework is transferable beyond cultured meat to general mammalian cell/tissue culture practice.

## 2 Physicochemical Basis of Osmolality and Measurement Nonidealities of Solutes in Complex Media and in Measurement Approaches

Osmolality (ξ) is the concentration of osmotically active solutes present in an aqueous solution, which is often expressed as mOsm/kg of the solvent by freeze point depression or as mOsm/L, known as osmolarity. For a medium containing multiple solutes, its theoretical osmolality can be expressed as^34^

1 $$\xi =\Sigma_{i}\varphi_{i}V_{i}m_{i}$$

where ξ is the osmolality (mOsm/kg of solution), *m_i_* is the molality (mol/kg of solution) of the solute *i*, *v_i_* is the number of particles formed by the dissociation of that solute *i*, and φ*_i_* is the molal osmotic coefficient (a measure of unity for a perfectly ideal, fully dissociated solute that does not bind with water or interact with other solutes) of the solute *i*.^34^ Generally, for a simple medium dominated by inorganic salts, the empirical rule “every 1 g of NaCl per liter of basal medium raises osmolality by ∼25 mOsm/kg” provides a useful approximation by assuming near-ideal behavior, and the “sum of osmoles” additive approximation holds relevance here.^2^

From a culture medium point of view, when the composition of solutes in a medium formulation is known (often the case for dilute or simple solution media), osmolality can be reasonably calculated using the theoretical eq 1. However, the inclusion of the osmotic coefficient (φ*_i_*) in this expression makes it clear that osmolality does not depend solely on solute concentration nor can it be attributed to the size, charge, or shape of any individual molecule.^35^ Instead, it emerges from collective solute–solute and solute–solvent interactions that intensify as medium complexity increases. The φ*_i_* quantifies how much a real solution deviates from ideal colligative behavior, and when solutes do not interact (ideal case), φ*_i_* ≈ 1, meaning that the theoretically calculated osmolality matches the measured osmolality. However, as the total number of osmotically active species and their combined physicochemical behaviors shape the effective osmolality, theoretical calculations begin to deviate systematically from the ideal colligative behavior, and φ*_i_* departs from unity. These deviations become significant, especially in complex formulations where diverse solutes ranging across inorganic salts, amino acids, sugars, zwitterions, proteins, polymers, etc., are present, which is the case for many contemporary chemically defined and serum-free mammalian culture medium formulations (especially for muscle cell cultures from a cultured meat point of view). For such complex media, experimental osmolality measurements often exceed theoretically calculated values because φ*_i_* would likely capture the nonideal behavior arising from these numerous interactions.

In practice, the osmolality of a medium solution is assessed using osmometers as a quality control metric, which operate on either freezing point depression (FPD) or vapor pressure depression (VPD) principles to infer ξ from colligative changes in solvent properties. Sahin et al. examined various protein/polymer solutes in medium formulations, revealing significant deviations between theoretically calculated (TC) osmolality and osmolality measured by osmometers using FPD and VPD principles.^34^ At high protein (>150 mg/mL IgG) and PEG 10K (0–22% w/v) concentrations, FPD osmometers tend to overestimate osmolality relative to theoretical calculations due to freezing-related artifacts such as cryoconcentration, phase separation, and protein–ice interactions in protein-rich media, as well as polymer-mediated reductions in water activity in PEG-containing systems. VPD tracked theoretical osmolality more closely but still deviated for strongly hydrogen-bonding polymers, including PEG, consistent with water activity measurements. Combined protein–polymer-centric formulations reduced water activity more strongly than either component alone (often PEG-only > IgG-only at similar mass fractions),^2,36^ reflecting extensive solute–water interactions that increase the effective osmolality of complex media (*the reader is referred to* Figure 1 *A,E in Sahin et al. (2016) for the impact of high-concentration proteins and polymers on apparent/measured osmolality using FVD and VPD osmometry*).^34^

**Figure 1:**
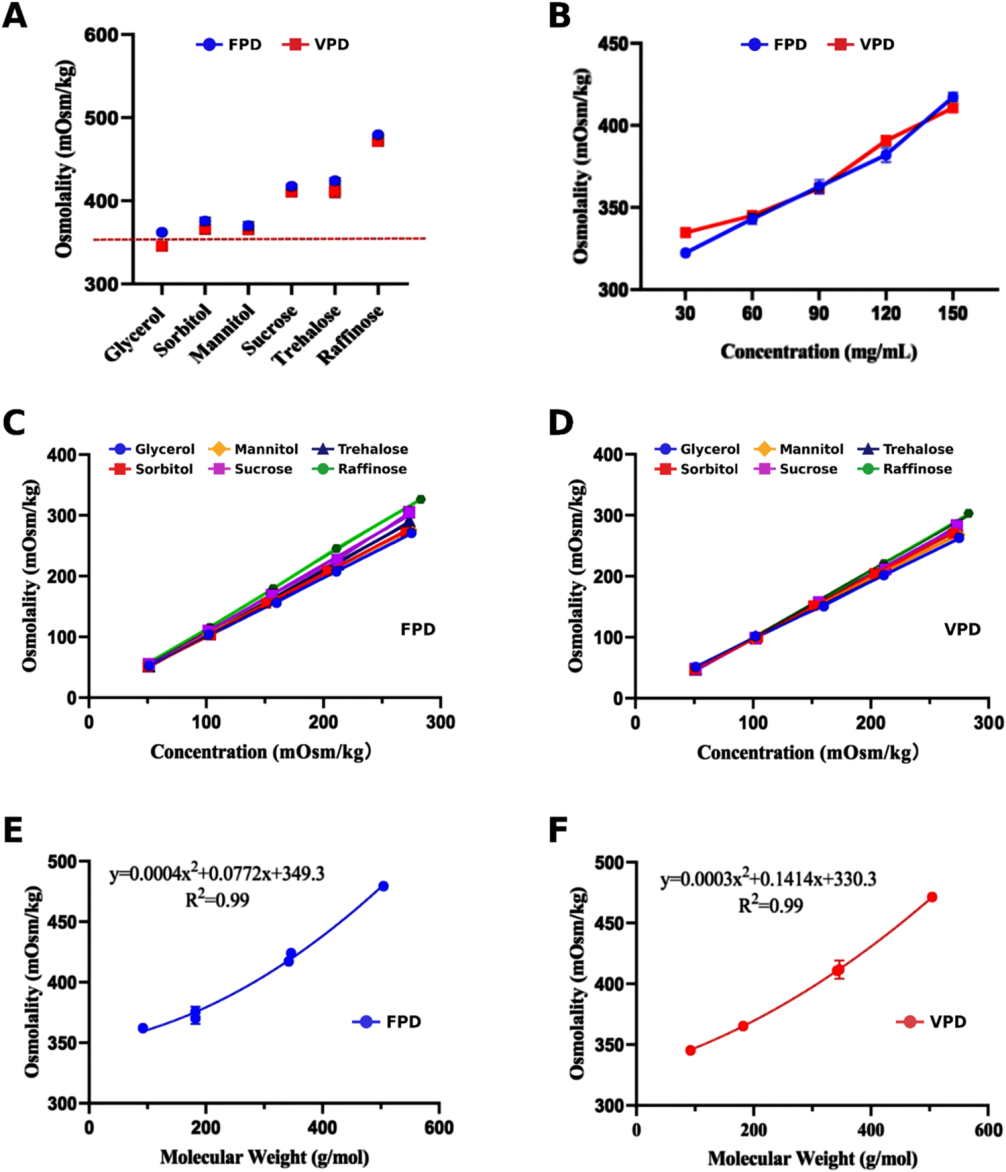
Quantitative results for nonideal osmolality behavior in high-concentration protein-centric formulations: (A) Bovine serum albumin (BSA) formulations containing different added excipients; measured osmolality from both FPD and VPD is consistently higher than the theoretical value (red dashed line), with glycerol (smallest molecular weight) showing the lowest and raffinose (largest) showing the highest. (B) Osmolality increased approximately linearly with BSA concentration by both FPD and VPD. (C) Osmolality of each excipient increased with concentration by both FPD and (D) VPD. Quadratic relationships (high *R* ^2^) between osmolality and excipient molecular weight by (E) FPD and (F) VPD. Modified and reprinted with permission from Pang, M. J., Habib, H., Wang, M. W.,& Fang, W. J. (2026). Nonideal osmolality behavior in high-concentration protein formulations: mechanistic insights from freezing point depression, vapor pressure deficit, and molecular dynamics simulations. *Int. J. Pharm.*, 126553. Copyright 2026, Elsevier.

Given the above case, Sweeney & Beuchat noted that the relationship between solute concentration and osmolality is not always linear and that the complex behavior of different solutes in solution again comes into play, a scenario that is often difficult to predict and understand theoretically alone and thus requires comprehensive empirical measurement.^35^ This was again demonstrated recently, in which high-concentration protein-centric formulations exhibited nonideal osmolality when ∼150 mg/mL bovine serum albumin (and similarly for monoclonal antibody A and lysozyme) was formulated with six excipients (glycerol, sorbitol, mannitol, sucrose, trehalose, raffinose). Both FPD and VPD osmometry measured the osmolality of these formulations, which consistently exceeded the theoretical prediction of ∼291 mOsm/kg, as shown in Figure 1A. The magnitude of this elevation depended strongly on the excipient structure, where, for instance, glycerol produced the lowest osmolality, while raffinose produced the highest, and the authors reported a quadratic relationship between osmolality and excipient molecular weight (*R* ^2^ ≥ 0.99) (Figure 1B,E,F). Mechanistically, molecular dynamics simulations indicated that excipients with more hydroxyl/ether groups strengthened solute–water hydrogen bonding and disrupted the water network, thereby aligning with the observed nonideal (synergistic) increases in osmolality when proteins and excipients coexist (Figure 1C,D).^37^ On the other hand, recent quantitative data show that osmolality drift during continuous culture depends not only on incubator humidification but also strongly on medium composition. Joris et al. reported that under humidified conditions, osmolality increased by only ∼4–6 mOsm/kg over 5 days in both simple and complex media.^121^ However, under nonhumidified conditions, a simple salt-based medium (SG-1) exhibited substantially larger increases (∼21–45 mOsm/kg) that scaled with starting osmolality, whereas a modern, compositionally complex medium (Gx-TL) showed more limited and largely starting osmolality-independent increases (∼19–23 mOsm/kg). Component-level analysis further revealed that the presence of amino acids or hyaluronan alone in the complex media reduced osmolality rise, with media containing both achieving the greatest stabilization (only ∼16 mOsm increase over 5 days),^121^ demonstrating that medium composition can act as an intrinsic osmotic buffer and that identical starting osmolality does not imply equivalent osmotic behavior during culture.

The rationality of the illustrated scenarios above could pose practical challenges for many contemporary bespoke culture media being developed, both in the use of diverse ingredients and in process quality control. For instance, two media may appear identical when measured with a single osmometer because they yield the same numerical osmolality. However, their underlying solute compositions and their various effects can be very different. As a result, they may impose distinct osmotic environments on cells despite appearing to be equivalent instrumentally. This challenge is further compounded if measurement practices, such as routine calibration of the osmometer against standard solutions, are not performed rigorously before analysis, as even modest instrumentation bias can obscure genuine formulation-dependent differences. For muscle lineage applications, which operate within a defined osmotic window, such discrepancies represent a key area for future research, particularly in understanding how osmolality-related measurement and formulation differences could propagate into changes in the cell phenotype, tissue fusion, and process robustness. These physicochemical nonidealities and measurement constraints directly determine the effective osmotic environment experienced by cells, thereby influencing the biological responses observed in mammalian muscle systems, as discussed in the next section.

## 3 Importance of Culture Medium Osmolality on Mammalian Muscle Cells/Tissues

Maintaining the culture medium osmolality within an isotonic, cell-tolerable range is essential because deviations from intracellular osmolality drive cell swelling or shrinkage, disrupting membrane transport, biosynthesis, and metabolism. While some mammalian cell types tolerate broad osmotic ranges, for example, L-cell fibroblasts, which showed consistent growth across ∼220–440 mOsm/kg of H_2_O to adjusted medium salt concentrations,^38^ muscle-lineage cells are typically more sensitive due to their structural specialization and cytoskeletal constraints, underdeveloped osmotic stress regulation mechanism (although this varies in degree with the cell line), and reliance on tightly regulated ionic gradients.^39,40^ General mammalian culture guidance often targets ∼260–320 mOsm/kg at pH ∼7.4 to approximate physiological serum osmolality (∼290 ± 30 mOsm/kg), but myogenic systems frequently operate within a narrower functional window, making osmolality control and its time-varied reporting particularly important for reproducible outcomes (Table 1).

These differences become clear when comparing common bioproduction tissue/cell types, such as robust lines, including CHO, which can tolerate hyper-osmolalities in the ∼400–450 mOsm/kg range and hence are often exploited for the enhanced antibody or recombinant protein production.^4,23,40–44^ Myoblasts and satellite cells typically perform best around 280–320 mOsm/kg, with declining proliferation and differentiation above ∼350 mOsm/kg.^11,45–47^ Consistent with this, skeletal muscle fibers are well-known to undergo osmotic volume adjustments in response to such extracellular osmolality (possibly revealing a threshold behavior that is likely cell line- and culture phase-dependent) to reach a state of homeostasis under such stress-inducing conditions.^48^ This contrast in different cell lines’ adaptation to differing osmotic conditions reinforces the need for the cell and its phase-specific osmotic design and measurement rather than relying on generic “isotonic” assumptions. As shown in Table 1, muscle-lineage cells have narrow osmolality tolerances among other mammalian cells.^7^ At the same time, fibroblasts, hybridomas, and especially chondrocytes often tolerate much broader windows, with chondrocytes even thriving under 350–450 mOsm/kg conditions consistent with their native hypertonic extracellular matrix environment.^49,50^

Various commercially available basal media add another layer of variability because their baseline osmotic loads are not equivalent. DMEM, for example, is often used for myogenic cultures and is formulated with comparatively elevated glucose and enriched amino acid and vitamin levels to support robust growth and consequently has a higher baseline osmolality. At the same time, EMEM and RPMI can differ by tens of mOsm/kg due to salt and buffer composition and vendor-specific QC ranges.^51–57^ In this context, muscle precursor cells (including commonly used myoblast models) can exhibit impaired proliferation and fusion competence when osmolality deviates from the physiological window as demonstrated by Herbelet et al. where increased medium osmolarity from baseline DMEM (∼280 mOsm/L) to hyperosmotic conditions (∼333–443 mOsm/L via NaCl) caused increased NFAT5 expression and nuclear translocation, indicating activation of an osmotic stress adaption cellular program.^7^ Functionally, NFAT5 is not merely a stress marker but also a muscle-intrinsic regulator of myogenesis; disrupting its transcriptional activity impairs myoblast migration and differentiation, thereby compromising subsequent myotube formation and fusion.^58^ Accordingly, NFAT5 provides a plausible mechanistic link between osmotic stress sensing and myogenic outcomes. However, NFAT5 translocation in this case should be interpreted as a cellular osmoadaptation to hypertonic stress and not as a stress-countering agent since its expression does not provide evidence that myogenesis outputs (differentiation/fusion) are preserved even under prolonged stress.

In fact, NFAT5-driven osmoadaptation has been shown in other proliferating mammalian cells to enable recovery of cyclin expression and cell cycle progression under hypertonicity, whereas NFAT5 deficiency is associated with persistent arrest, offering a possible mechanistic route by which prolonged osmotic excursions could limit myoblast expansion and compromise subsequent fusion competence when cultures operate outside the optimal osmotic window.^59,60^ Finally, NFAT5 is not exclusively tonicity-dependent, and signals such as cytokines, growth factors, receptor/integrin activation, contractile agonists, ions, and reactive oxygen species can also regulate its expression and activity.^61^ Therefore, NFAT5 translocation should be interpreted in this context as supporting osmotic stress engagement in a hypertonic experiment but not serving as a universal, tonicity-specific readout across all culture conditions.

Beyond activating stress-response pathways, hyperosmotic conditions initially induce rapid water efflux and acute cell shrinkage, leading to increased intracellular macromolecular crowding and a shift of the cytoplasm toward a more crowded, diffusion-limited physicochemical state with constrained molecular motion and altered reaction kinetics.^33,62–64^ Hypo-osmotic conditions, conversely, promote cell swelling and perturb membrane transport processes and signaling cascades that depend on tightly regulated ion gradients and volume homeostasis.^65–67^ In skeletal muscle and other mammalian cells, such initial hypertonic shrinkage to external hyperosmotic conditions triggers a regulatory volume increase (RVI), wherein inward solute accumulation (predominantly mediated by Na^+^–K^+^–Cl^–^ cotransporter (NKCC) activity) together with associated cation and anion transport pathways increases intracellular osmotic content and thereby promotes passive water re-entry and partial volume recovery over time scales of tens of minutes to several hours.^48^ In muscle cells, for example, both the magnitude and kinetics of this recovery are strongly fiber type-dependent, with slow-twitch fibers exhibiting a more rapid and more complete RVI than fast-twitch fibers under equivalent hyperosmotic challenge.^48^ Consequently, skeletal muscle cells do not follow ideal osmotic behavior and, as emphasized by Lindinger et al., “*mammalian skeletal muscle cells don’t always behave as a perfect osmometer*,” meaning that cell volume changes are not simply proportional to extracellular osmolality but instead display nonlinear, fiber-specific deviations shaped by transporter activity, cytoskeletal constraints, intracellular osmolyte handling, and membrane–cytoplasm mechanical coupling.^48,68^

When hyperosmotic exposure is sustained over longer durations (hours to ∼24 h), volume regulation increasingly transitions from ion flux-dominated recovery toward slower adaptive processes involving transcriptional, metabolic, and structural remodeling, most notably through activation of tonicity-responsive factors such as NFAT5/TonEBP.^107^ Experimental measurements using intracellular crowding sensors and live-cell approaches demonstrate that apparent restoration of cell volume at these later time points does not necessarily correspond to recovery of the original intracellular physicochemical state but rather reflects establishment of a newly adapted steady state characterized by persistent alterations in crowding, ion distribution, and molecular mobility.^108,109^ Complementing these observations, mechanistic and mathematical modeling frameworks explicitly show that cell volume dynamics unfold across separable fast and slow time scales where there is an initial phase dominated by rapid water and anion fluxes followed by a slower phase constrained by sodium-coupled transport and membrane potential adaptation, thereby explaining why late-time measurements of cell volume, ion-coupled volume recovery, and intracellular crowding state obtained after 12–24 h can differ qualitatively from initial postshock volume changes and instead reflect regulatory adaptation rather than acute osmotic responses.^110,111^ Collectively, these time-dependent and nonideal volume-regulatory behaviors imply that even modest extracellular osmolality shifts could potentially influence the kinetics and efficiency of key myogenic outcomes such as proliferation rate, differentiation efficiency, and myoblast–myotube fusion events, particularly when those osmotic perturbations related to cellular activities coincide with these specific cellular phases, thereby reinforcing the need for time-resolved interpretation of osmolality–phenotype relationships in muscle cell systems.

In this context, it is important to emphasize that changes in cell volume observed during these later adaptive phases should not be interpreted as direct indicators of subsequent progression of normal proliferative or mitotic activities. Across multiple mammalian cell lines, osmotic and related physicochemical perturbations have been shown to dissociate volumetric adaptation from cell cycle advancement, which instead manifesting as prolonged residence of cells in *G*_0_/*G*_1_, delayed or suppressed entry into the S-phase, and in some systems an increased fraction of cells arrested at the *G*_2_/M checkpoint as part of stress adaptation. For example, single-cell and population-level studies in human epithelial and carcinoma-derived cell lines (including MDA-MB-231 breast cancer cells and A549 lung carcinoma cells) demonstrate that hyperosmotic stress induces reversible growth arrest and *G*_2_/M delay despite apparent recovery of cell volume and nuclear size.^112,113^ Such phase-specific cell cycle effects can coexist with apparent recovery or stabilization of cell volume, underscoring that volume recovery primarily reflects regulatory and stress-adaptive state transitions rather than progression through the cell cycle. While these behaviors are increasingly being characterized in nonmyogenic mammalian cell lines, how osmotic volume regulation intersects with differentiation-associated exit from the cell cycle (i.e., stable withdrawal into *G*_0_/*G*_1_) and with the activation, maintenance, and resolution of classical *G*_1_/S and *G*_2_/M checkpoints in muscle-lineage cells remains incompletely resolved and warrants further targeted investigation. Given these cell-specific sensitivities, controlling osmolality becomes not only a biological requirement but a formulation challenge, necessitating a framework for understanding how medium composition defines the accessible chemical space as discussed in the next section.

## 4 Media Composition, Chemical Space, and Strategies for Osmolality Control

Designing an effective culture medium requires balancing nutritional requirements with a cell/tissue’s physicochemical stability. Each solute in a formulation, be it salts, amino acids, sugars, buffers, vitamins, or various novel supplements, contributes additively to osmolality. Still, the collective interactions among solutes and their relative concentrations determine the total osmotic environment experienced by cells.

### 4.1 Contribution of Individual Components

Typical basal media differ considerably in osmotic strength, as shown in Figure 2A, which depicts the measured values for standard formulations being ∼330 mOsm/kg (DMEM), 290–300 mOsm/kg (EMEM), and ∼310 mOsm/kg (IMDM).^51–57^

**Figure 2:**
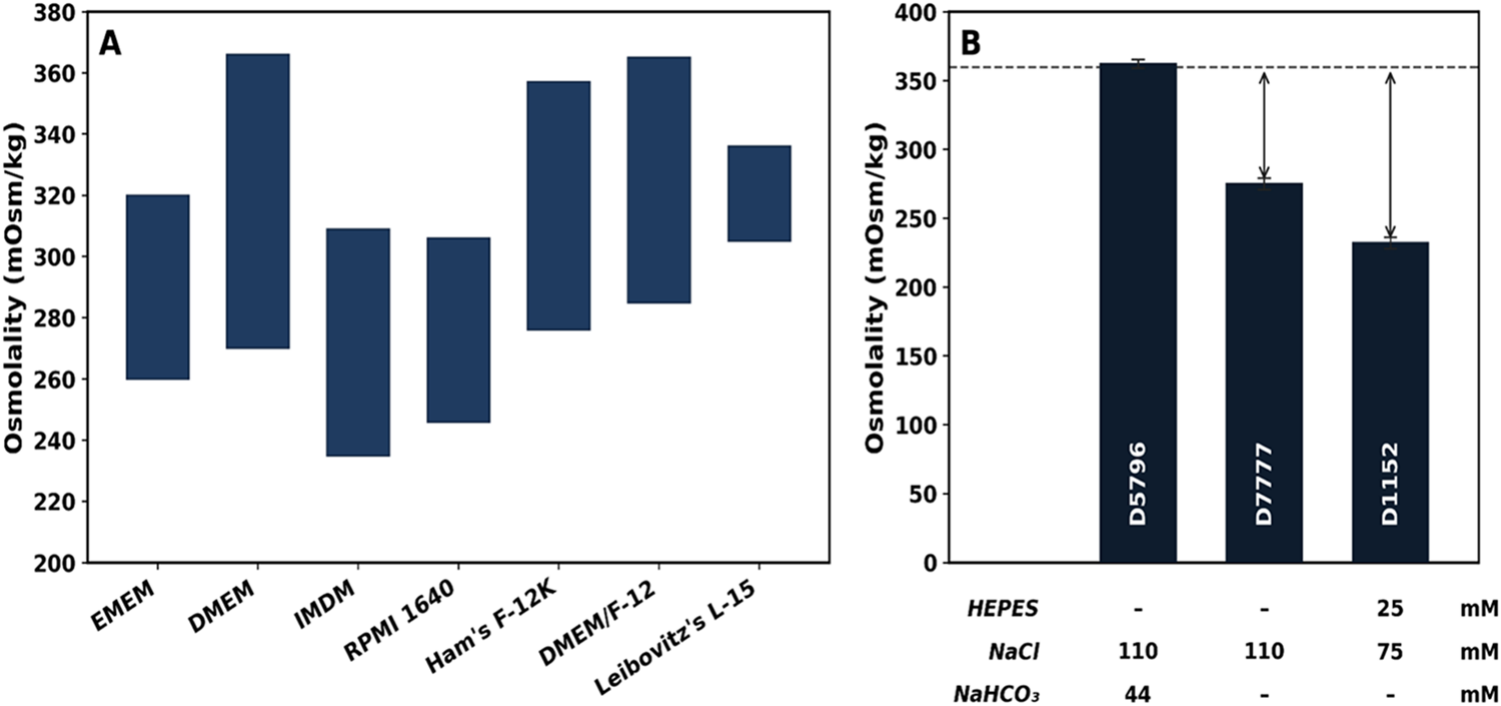
(A) Osmolarity range of commonly used basal media including EMEM, DMEM, IMDM, RPMI 1640, Ham’s F-12K, DMEM/F-12, and L-15. Values taken from the ATCC product sheet.^51–57^ (B) Osmolality of three different medium formulations, where the arrow indicates the chemical space available in HCO^3–^ buffer-free media, which offers an osmotic gap for the addition of other osmolytes (HEPES, NaCl, NaHCO3), which can be customized based on the cell line to be cultured, regardless of eventually reaching physiologically relevant osmolality. Note: While this could still be used to maintain preferred osmolality, one has to still keep an eye on the pH dynamics of the culture media before and after the addition of osmolytes, especially in the case of nonvolatile buffers, due to CO_**2**_–HCO_**3**_^**–**^ equilibrium, since during CO_2_ incubation, the use of nonvolatile buffers instead of HCO_**3**_^**–**^ buffer will cause the media to become acidic. Reprinted with permission (CC-BY) from Michl, J., Park, K. C.,& Swietach, P. (2019). Evidence-based guidelines for controlling pH in mammalian live-cell culture systems. *Commun. Biol.*, 2(1), 144. Available under a CC BY 4.0 license. Copyright 2019, Springer Nature.

For multisolute solutions, osmolality depends on the number of dissolved particles each solute/ingredient generates (“effective particle count”) and not simply the mass added, since dissociation increases the number of osmotically active species.^69^ Thus, salts and amino acids, when present as ionic species or supplied as salts, typically contribute more osmoles per mole than nonelectrolytes, since their dissociation generates multiple particles in solution.^69^ Consistent with this, direct measurements of water activity/osmotic coefficients show distinct solution behavior for neutral amino acids (e.g., glycine) versus ion-containing amino acid salts (e.g., sodium glutamate), reflecting the larger “effective particle” contribution of ionic forms.^70,71^ By contrast, most carbohydrates (e.g., glucose, sucrose) are nonelectrolytes (van’t Hoff factor ≈ 1) and therefore add fewer particles per mole. Moreover, sugars hydrogen-bond strongly with water, altering water activity and other solution properties without generating additional ions.^72^ Amino acids themselves can also behave as dipolar ions (zwitterions) in aqueous solution, and many also have ionizable side chains.^73^ From a medium design standpoint, the practical implication is that osmolality can often be reduced without removing nutritional functionality by reallocating solutes, for example, minimizing unnecessary inorganic salts or choosing more weakly dissociating ion forms of supplements wherever feasible, while still maintaining the required amino acids and other essential molecules/compounds. These component-level contributions therefore define how quickly the available osmotic budget is consumed, motivating the need for a more explicit and operational framework for managing compositional limits within this “chemical space” as explained in the next sections.

### 4.2 Expanding Chemical Space without Exceeding Osmotic Limits

The concept of “chemical space” can be defined here as the allowable compositional range within which medium components can be added while maintaining total osmolality within a functional window for a given cell type and culture phase. In this framework, osmolality represents a hard upper constraint: each added solute contributes to the total osmotic load and therefore “consumes” part of a finite osmotic budget.

As discussed earlier, from a medium optimization point of view, nutrients must be supplemented so that final osmolality remains within the cell line-specific optimum (typically close to common basal medium ranges) osmolality range because growth inhibition and an altered phenotype can occur when cultures operate outside the optimum window. In most cases, the optimum operating range is determined empirically by testing different formulations and component levels supplemented to a common basal medium. Conceptually, medium components can be grouped into (a) “consumables” (glucose/energy sources, growth factors, amino acids, proteins, vitamins, added secretomes, ligands etc.) and (b) “nonconsumables” (inorganic salts and buffers) based on components that cells take up and metabolize (so their concentration tends to decrease over time) and those that maintain the physicochemical environment of the medium, respectively.^74^ In many formulations, as expected, nonconsumables will dominate baseline osmolality because they dissociate into ionic species, which subsequently contribute strongly to the particle count. Although consumables, on the other hand, often contribute less to osmolality at typical working concentrations, they can still drift osmolality upward later in culture when they are densely fortified in a medium, since a high nutrient load will drive subsequent metabolite accumulation (e.g., lactate/ammonia) and ion imbalances (Na^+^/K^+^), resulting in an increase in the total dissolved osmoles.^75^

A simplified categorization of common medium components and their relative contributions to osmolality is shown in Table 2, highlighting that chemical space is not only a quantitative constraint but also a compositional one. Within this framework, “consumption” of the chemical space is primarily driven by highly dissociating or high-concentration solutes such as salts, buffers, and accumulated metabolites. Conversely, strategies that “free up” chemical space include reducing ionic strength where possible, replacing high-osmolality components with lower impact alternatives, using more efficient buffering systems, and incorporating macromolecular additives that provide functional benefits (e.g., stabilization or crowding) with comparatively low osmotic contribution.^49,76,77^ This is illustrated by some commonly explored strategies:

(i) Substituting part of the CO_2_/HCO_3_^–^ buffer system with zwitterionic buffers (e.g., hydroxyethyl piperazineethanesulfonic acid/3-(N-morpholino)­propanesulfonic acid) to maintain pH control while reducing dependence on bicarbonate chemistry that can rather introduce additional ionic load and produce nonintuitive pH outcomes if not managed carefully.^3^ For instance, media formulations without HCO_3_^–^ buffer such as D7777 (NaHCO_3_-free DMEM from Sigma-Aldrich) offer chemical space for addition of other osmolytes (a total of 88 mOsm/kg was added for culturing) such that the final osmolality is the same as ready-to-use commercial D5796 (Sigma-Aldrich) used for culturing Caco2, DLD1, and NCI-H747 cells shown in Figure 2B.^78^
(ii) Many inorganic ingredients in basal media (salts, buffers, certain ions) mainly exist to maintain physicochemical conditions rather than to be consumed by cells. Because they often dissociate into multiple ions, they can take up a large share of the medium’s “chemical space”. If some of these components are redundant (not needed for the specific cell line/process) or present in excess, removing or reducing them frees up “chemical space” to which other functional components can be added. This was demonstrated in perfusion medium, where unnecessary components were removed, and osmolality was explicitly optimized (e.g., a ∼360 mOsm/kg feed medium yielding ∼300 mOsm/kg residual culture osmolality).^79^
(iii) Introducing “isotonic enrichers”, i.e. adding large, nonelectrolyte molecules (e.g., albumin or neutral polymers) to make the medium feel more tissue-like. This increases the viscosity and macromolecular crowding without greatly increasing osmolality because these large molecules contribute relatively few dissolved particles at typical working levels. This can help tune extracellular matrix organization and cell behavior and could support the design of custom media that deliberately introduce macromolecular crowding effects in the cultured tissue (discussed next).^80^

**Table 2:** Common Medium Components and Their Relative Contributions to Osmolality

category	impact on osmolality	key considerations
salts (e.g., NaCl, KCl)	high	fully dissociate into multiple ions, strongly increasing osmotic load
buffers (e.g., bicarbonate, HEPES)	medium–high	contribute depending on the concentration and pH control strategy
amino acids	medium	contribution varies with the form (zwitterionic vs charged) and concentration
small metabolites (e.g., glucose)	medium	nonionic but present at relatively high concentrations
waste products (e.g., lactate, ammonia)	medium–high	accumulates over time and drives osmotic drift
macromolecules (e.g., albumin, polymers)	low (per mass)	large molecules often have a low molar contribution; they may contribute to crowding rather than to osmolality

These principles become particularly salient in the design of bespoke serum-free/chemically defined muscle-cell media, where serum functionality must be reconstructed using defined additives (growth factors, carrier/stabilizing macromolecules). This typically increases the number and concentration of solutes, which can push formulations beyond the available “chemical space” unless the ionic composition is deliberately controlled.^12,74,75,82^ In parallel, the broader push toward serum-free, protein-free, animal-derived component-free and chemically defined media has driven extensive trials in which basal media are fortified with defined proteins and supplements (e.g., serum albumin, fetuin, insulin, ITS, dexamethasone, fibronectin/vitronectin, fatty acids such as α-linolenic acid, and signaling modulators such as IL-6 and curcumin), as well as extracts (e.g., microalgal extracts, silk sericin) and recombinant growth factors/hormones (e.g., TGF-β family factors, IGFs, PDGFs, bFGF/FGF-2) to support myogenic proliferation, differentiation, and other performance attributes.^10,11,47,76,77,81,82–85^ For context, in cell culture terminology, “serum-free” denotes the absence of whole serum (e.g., fetal bovine or horse serum) but does not preclude the use of purified proteins such as albumin. In contrast, formulations lacking even such protein supplementation are more appropriately described as “protein-free”. Taken together, the above insights suggest a need to understand better how medium components and their interactions influence the overall ionic balance, osmotic pressure, and signaling environment in muscle-cell cultures. Addressing these questions will require systematic studies that link medium composition with key cellular and physicochemical outcomes. Such work could establish clearer design principles for serum-free and chemically defined media, improving both reproducibility and functional control across applications. In this context, the concept of chemical space provides a practical basis for translating compositional constraints into real-world medium design strategies tailored to myogenic systems.

### 4.3 Implications for Myogenic Medium Design

Conventional serum-containing media provide broad trophic, stabilizing, and buffering functions that support robust cell growth but rely on compositionally variable, undefined components. Replacing these complex mixtures with serum-free, chemically defined formulations, therefore, requires careful tuning of both biochemical and physicochemical parameters to maintain comparable functionality and reproducibility. Recent work illustrates this progress where, for example, the chemically defined B8 medium was optimized through systematic tuning of medium composition and explicit control of pH and osmolality, achieving maximal growth of human induced pluripotent stem cells at ∼290–310 mOsm/L and pH ∼7.0–7.1, while supporting long-term culture (>100 passages) at an estimated reagent cost of ∼US$16 L^–1^ using in-house recombinant proteins.^86^ In the cultivated meat context, Beefy-9 adapts a B8-derived base for bovine satellite cells by adding recombinant albumin at 800 μg/mL (0.8 g/L), enabling sustained expansion over seven passages with an average doubling time of ∼39 h but requires a defined workflow (e.g., delayed albumin addition and optimized adhesion coatings) to maintain robust attachment and growth.^10^

More recent studies indicate that albumin-based medium stabilization and replacement strategies are strongly species- and cell line-dependent. For example, B9-type formulations for bovine satellite cells typically incorporate ∼0.8 g/L human serum albumin (HSA), which has been estimated to contribute ∼US$24.56 L^–1^ to medium costs. In contrast, other serum-free bovine myoblast culturing media have employed substantially higher albumin concentrations (up to 5 mg/mL (5 g/L)) in chemically defined formulations that substitute bovine serum albumin (BSA) for HSA during development. Although lower cost excipients such as methylcellulose (∼0.1125 g/L) and alanine (5–20 mM) can partially substitute albumin’s stabilizing role or act synergistically with HSA in specific formulations, their effectiveness is not uniform across cell types. In particular, the same studies report divergent growth and differentiation outcomes when applying these albumin reduction strategies to bovine, porcine, and chicken satellite cells, with distinct responses again observed in CHO cells, despite cost reductions of up to ∼73% being achievable for selected cell lines.^86,87^ Collectively, these studies emphasize that the development of myogenic media must integrate osmotic control, macromolecular stabilization, and species-specific growth requirements into a defined and testable framework. Advancing this field will require systematic, multiparameter investigations that couple quantitative proteomics, osmotic stress response assays, thermodynamic and colloidal modeling of particulates, and their ionic interactions. Such systematic, cross-comparative investigations into molecular function, excipient synergy, and ionic balance could generate predictive rules linking formulation chemistry to cellular performance, enabling more reliable and transferable design of next-generation serum-free and chemically defined muscle-cell media.^87^

Consistent with this strong cell and species dependence, several groups have explored “low-albumin” serum-free formulations that minimize total protein content while including a focused set of defined supplements and growth factors. For instance, Skrivergaard et al. developed an in-house serum-free medium based on DMEM/F12, supplemented with FGF2, fetuin, BSA (75 μg/mL), ITS, and a custom platelet-derived growth factor variant, to compare satellite cell proliferation between bull calves and dairy cows under both serum-free and serum-containing (10% FBS) conditions.^45,88^ In these culture studies, bull-calf satellite cells exhibited significantly higher proliferation than dairy-cow cells under both serum-free and serum-containing conditions, while subsequent differentiation and myotube formation were broadly similar across donor types. Notably, independent of donor origin, the serum-free medium enhanced proliferation relative to 10% FBS, delayed spontaneous differentiation, and yielded higher final cell densities with reduced interanimal variability.

Recently, coculture strategies have been reported in myogenic and cultivated meat research to better reproduce native cellular heterogeneity, paracrine signaling, and tissue-level organization, particularly through muscle–adipose and multilineage systems.^114,115^ In practice, these platforms rely heavily on conventional mammalian basal media, most commonly DMEM or DMEM/F-12-derived formulations supplemented with serum, growth factors, or food-grade additives, and are implemented in static plates, Transwell systems, or three-dimensional scaffold-free cultures rather than stirred bioreactors.^114,116^ Recent cultivated meat studies exemplify this, specifically using coculture and codifferentiation of muscle stem or satellite cells with adipose precursor or mesenchymal cells to optimize simultaneous myogenic and adipogenic differentiation, thereby promoting intratissue lipid accumulation and early marbling-like features relevant to meat texture and sensory attributes. These systems employ serum-free or reduced-serum DMEM or DMEM/F12-based media and tune differentiation primarily through lineage-specific induction cues. In practice, this involves staged or parallel exposure to myogenic and adipogenic differentiation media, temporal modulation of myogenic versus adipogenic induction, and in some systems adjustment of the relative proportions of muscle stem/satellite cells and adipose precursor cells to balance myotube formation with lipid accumulation and adipogenesis.^117,118^ However, these studies also introduce additional sources of osmotic complexity through conditioned-medium effects, secretion of extracellular matrix components, accumulation of metabolic byproducts, and the concurrent activity of multiple cell populations with distinct nutrient uptake and ion-handling behaviors.^119^ Notably, despite this increased biochemical and metabolic load, explicit measurement or reporting of final working osmolality is generally absent. As commonly used DMEM and DMEM/F12 formulations already operate near the upper end of physiological osmolality, such coculture configurations are intrinsically more susceptible to time-dependent osmotic drift and the emergence of locally heterogeneous osmotic microenvironments, even when bulk medium composition appears nominally unchanged.

Taken together, these observations underscore that the performance of serum-free myogenic media remains highly sensitive to the formulation and cell origin. However, across both advanced monoculture and emerging coculture strategies, many customized or optimized formulations still omit explicit reporting of the final working osmolality, limiting cross-study comparability and complicating efforts to decouple the relative contributions of biochemical composition and the osmotic environment to observed myogenic growth, differentiation, and maturation outcomes.

Building on these concepts, macromolecular crowding (MMC) offers another biomimetic strategy that better replicates the macromolecule-dense environment of native muscle tissue. By adding high-molecular weight “crowders” such as Ficoll or dextran polymers, MMC leverages the excluded-volume effect (where occupied space by large polymers reduces the free volume for other macromolecules, effectively increasing their local concentration) to promote effective macromolecular interactions and enhance extracellular matrix (ECM) assembly and organization.^89^ For example, Zeiger et al. demonstrated that crowded media improved ECM alignment and actin organization in myogenic cultures, illustrating how medium biophysics can direct cell–matrix reciprocity.^80^ Such effects are particularly relevant to muscle-cell models (C2C12, primary myoblasts, bovine satellite cells), where ECM alignment governs the fiber structure and tissue organization, which are key parameters for cultivated meat applications. MMC can be implemented within serum-free or chemically defined media using neutral or charged, food-grade polymers such as dextran.^90^ However, careful osmolality control is essential, as excessive crowding can cause hypertonicity-induced cell shrinkage and obscure genuine biophysical effects induced by increased intracellular MMC (isotonic medium plus 600 mM mannitol) in NIH/3T3 fibroblasts.^91^ To fully capture MMC’s potential, future work should include quantitative mapping of crowding and osmolality interactions, integrating osmometry, rheology, and live-cell imaging with modeling of volume regulation and protein–polymer thermodynamics.

Embedding these analyses within process-scale studies, which would account for mixing, oxygen transfer, and shear, can likely enable MMC to function as a tunable design variable rather than a simple additive. Such system-level investigations will strengthen the rational, osmolality-aware, and cell-specific medium design framework needed for scalable, physiologically representative muscle cell culture and cultivated meat production. However, even optimized formulations do not remain static during culture, and their effective osmolality is further shaped by dynamic process-driven factors that evolve over time.

## 5 Physical, Chemical, and Material Factors Governing Osmolality Drift and Their Biological Implications for Muscle-Cell Culture

Even when culture media are initially formulated to the physiological range, experimental osmolality frequently increases or decreases during incubation, with the most important contributors being the pCO_2_ and buffer chemistry, pH control, temperature fluctuations, evaporative water loss in small volume culture, and medium interactions with the scaffold material often used for seeding cells, among others. Across a week-long run, combined drift mechanisms can shift osmolality by tens of mOsm/kg, where pCO_2_/pH coupling alone can increase osmolality by ∼27–51 mOsm/kg in bioreactors, and evaporation in low-volume cultures can add ∼20–30+ mOsm/kg over ∼6 days.^40,92,93^

### 5.1 pCO_2_, pH Regulation, and Its Osmolality Coupling

Most basal media (e.g., DMEM, IMDM) rely on the physiological CO_2_/HCO_3_^–^ buffering system, which inherently couples pCO_2_, pH, and osmolality. CO_2_ is not a metabolic requirement for most mammalian cultures; rather, it is supplied to dissolve in the medium, where a fraction hydrates to form carbonic acid (H_2_CO_3_), which then dissociates into bicarbonate (HCO_3_^–^) and H^+^, thereby controlling pH via equilibrium shifts (Le Chatelier behavior). Because osmolality scales with the number of dissolved particles (ions and molecules), any process that increases bicarbonate and associated counterions increases the total osmotic particle load and therefore tends to raise measured osmolality. In practical terms, the bicarbonate concentration in the basal medium dictates the required CO_2_ set point and the sensitivity of pH to pCO_2_ drift. For example, common media are often formulated around ∼26 mM NaHCO_3_, whereas DMEM is frequently formulated with much higher bicarbonate (∼44 mM), making its pH/osmotic state more tightly coupled to CO_2_ handling.^78,32^ This coupling becomes a hidden mechanism for osmolality drift under routine pH control and during scale-up. When pCO_2_ rises (e.g., with high cell density, due to limited stripping or high pressure), pH tends to fall. In contrast, if pH is actively maintained (e.g., by base addition), the “correction” effectively preserves or increases the bicarbonate/ionic load, so osmolality can rise even if pH appears stable. Classic bioreactor studies illustrate this magnitude, where, for instance, Kimura & Miller reported that elevating pCO_2_ from ∼4.8 to 33.3 kPa (∼36 to 250 mmHg) increased the measured osmolality by ∼25 mOsm/kg and reduced CHO growth by ∼45%.^40^ deZengotita et al. further showed that because bicarbonate equilibrates with CO_2_, higher pCO_2_ at constant pH produces a proportional osmolality increase, and growth inhibition can be substantially worse when such osmolality is not compensated (e.g., at ∼26.0 kPa (195 mmHg), ∼45% growth reduction with partial osmolality compensation to 361 mOsm/kg versus ∼63% reduction at 415 mOsm/kg without compensation).^22^ In CHO cell line culture studies, Zhu et al. also reported that raising pCO_2_ from ∼6.7 to 20.0 kPa (50 to 150 mmHg) at controlled osmolality (∼350 mOsm/kg) reduced specific growth rate, while independently increasing osmolality from 316 to 445 mOsm/kg caused a large growth rate drop (reported as ∼60% over that range), underscoring that CO_2_ stress and osmolality stress can be separable but often co-occur at scale.^23^

Although these quantitative examples come from other bioindustrial cell lines, the implications apply to myogenic cell lines as well, since a CO_2_-driven rise in the order of “tens of mOsm” can push cultures across a functional threshold. For serum-free and medium-dense formulations, where additional supplements already consume the “chemical space”, this pCO_2_–bicarbonate coupling reinforces why osmolality should be measured and reported alongside pH, rather than being inferred from nominal medium recipes or incubator settings. Proteome-level changes and cellular mechanistic responses to pCO_2_ at normal (37 °C) and reduced (33 °C) culture temperatures have also been reported.^106^ In addition to gas–buffer interactions, temperature introduces further complexity by influencing both osmolality measurement and the chemical stability of medium components.

### 5.2 Temperature-Linked Osmolality Drift: Implications for Measurement, Medium Design, and Myogenic Cultures

Temperature influences culture medium osmolality in two coupled ways: (i) it changes the apparent osmolality reported by osmometry (because osmolality is a thermodynamic colligative property expressed with an explicit *RT* term and nonideality via osmotic coefficients) and (ii) it accelerates chemical transformations of labile medium components that add new osmolytes over time. In practice, routine freezing point depression and vapor pressure osmometers have method-specific assumptions and limitations, so media measured at nonstandard temperatures (e.g., chilled 4–20 °C) can read systematically lower than the same medium at culture temperature; therefore, osmolality control limits should be defined using a standardized measurement temperature and clearly reported method (FPD vs VPD).

In parallel, thermally induced chemical drift can raise osmolality over time, where, for instance, common medium components like l-glutamine will spontaneously degrade into ammonia and pyrrolidone carboxylic acid, and in 48 h at 37 °C, these byproducts can increase osmolality by ≈12 ± 4 mOsm/kg and can generate >0.6 mM ammonia, a level sufficient to impair myoblast energetics.^2^ Accordingly, best practice is to standardize the timing/temperature of osmolality measurements and minimize glutamine breakdown by using fresh preparation or stabilized dipeptide substitutes (e.g., alanyl-glutamine).^94^ Temperature is also a formulation design variable because it determines the solubility limits of many medium components to which end, Vermeire et al. provide an open-source predictive framework (and associated SolProp dataset) that estimates the solid solubility of neutral organic solutes in water and other diverse organic solvents across a wide temperature range using molecular identifiers (e.g., SMILES/InChI), enabling potential for rational screening of supplements that remain soluble at the intended working temperature (e.g., 37–39 °C) and reducing the risk of precipitation-driven composition drift in many novel culture media.^95,96^

Temperature is not only a stability variable but can also be deliberately used as a biological cue (e.g., mild hyperthermia). In C2C12 and related myogenic models, Lee & Choi showed that exposure to 39 °C (intermittent or continuous) promoted proliferation and enhanced differentiation in a time-dependent manner, increasing myotube size and fusion index alongside upregulation of myogenic and mitochondrial markers (e.g., myogenin and PGC-1α-linked programs).^46^ Complementary work further supports temperature-sensitive myogenesis in C2C12, where controlled heat stress at 39 °C enhanced myofibrillogenesis and increased the expression of key myofibrillar genes/proteins relative to 37 °C controls. Critically, temperature and osmolality can interact rather than act independently, as Laitano et al. demonstrated in isolated mouse muscles, where isotonic hyperthermia (41 °C at 285 mOsm/kg) increased resting tension and injury/oxidation readouts in fast muscles. In contrast, modestly hypertonic hyperthermia (41 °C at 300 mOsm/kg) attenuated sarcolemmal injury and protein carbonylation, indicating that relatively small osmolality shifts can modulate heat stress outcomes.^97^ Taken together, these findings motivate a translational hypothesis for cultivated meat bioprocessing that if myogenic cultures (e.g., satellite cells and myoblasts) are operated within a carefully defined osmotic window, mild hyperthermia could potentially be leveraged as a controllable process cue to sustain proliferation and/or enhance differentiation while avoiding unintended injury-like stress, thereby underscoring the need to treat temperature and osmolality as coupled design variables when developing structured muscle-tissue processes. Beyond these thermodynamic and chemical effects, physical water loss through evaporation provides a direct and often dominant mechanism for the osmolality increase, particularly in small-scale systems.

### 5.3 Evaporation-Linked Osmolality Drift

Evaporative concentration is one of the main causes of osmotic rise in small-volume cultures, where the surface area-to-volume ratio is high. Even minor water loss can significantly increase medium osmolality: a 1% decrease in volume raises osmolality by roughly 3 mOsm/kg, while a 5% loss increases it by about 15 mOsm/kg.^92^ Wiegmann et al. quantified this effect in microscale systems, showing that evaporation-driven concentration can be monitored by increasing Na^+^ levels and mitigated through periodic water replacement, thereby highlighting evaporation as a practical driver of osmotic drift.^21^ In multiwell plates, evaporation is often spatially uneven, with edge wells and other high “evaporation index” positions experiencing greater water loss, leading to heterogeneous osmolality across a single plate unless humidity or plate sealing is carefully controlled. Consistent with this, Chi et al. observed osmolality increases exceeding 20 mOsm/kg within 24 h in dry incubators for IVF media.^98^ Similarly, Swain et al. found that small drop volumes and elevated setup temperatures can synergistically amplify osmotic rise, causing shifts large enough to impair embryo- and cell-level outcomes.^92^ Broader analyses by Mestres et al. further identified incubator humidity, oil overlay, medium composition, and dish geometry as primary determinants of evaporation and osmolality stability.^99^ Media starting at higher osmolality are particularly prone to reaching hypertonic levels as evaporation progresses.

Although evaporation effects are most pronounced in small-volume systems, they are not limited to them. Kimura & Miller reported ∼25 mOsm/kg osmolality increases over 1 week in aerated flask and bench-scale CHO cultures lacking active gas humidification, showing that water imbalance remains relevant even at larger culture volumes. Importantly, evaporation not only raises osmolality but also concentrates buffers and solutes, intensifying dissolved-gas and pH fluctuations through altered gas–liquid equilibria.^40^ Maintaining incubator humidity ≥95% or using condensation-sealed plates can limit daily osmolality changes to ≤0.5 mOsm/kg, preventing osmotic stress during long-term myogenic differentiation (>7 days). In bioreactors, humidified gas feeds or condenser coils provide comparable control, ensuring water balance and osmotic stability across extended culture durations.

In stirred-tank bioreactors for myogenic cultures, evaporation is effectively controlled through humidified gas feeds that saturate inlet air/O_2_/CO_2_ to 100% relative humidity, condenser coils in the exhaust line to recapture water vapor, and headspace minimization via vertical impellers and tight seals on single-use bags. Periodic sterile water additions, guided by Na^+^ monitoring, correct residual losses, while optimized low-shear agitation and jacket cooling reduce evaporative flux without compromising oxygen transfer. These strategies collectively maintain osmolality stability (<0.5 mOsm/kg daily drift), safeguarding proliferation, myotube formation, and differentiation across scales ranging from flasks to production bioreactors.^100,101^ While evaporation alters bulk osmolality, interactions with biomaterials introduce an additional layer of spatial complexity by generating localized osmotic microenvironments at the cell–material interface (see the next section).

### 5.4 Biomaterial Scaffold and Its Impact on Medium Osmolality

Biomaterial–culture medium interactions can introduce ions and small molecules from scaffolds, potentially shifting osmolality either in the bulk medium or locally at the scaffold surface. Scaffolds are critical in myogenic culture, especially for cultivated meat, as they provide 3D structural cues that guide myoblast/satellite cell alignment, fusion, and maturation into tissue-like constructs, while enabling scalable product shaping. However, biomaterial degradation (e.g., hydrolysis of biodegradable polymers) or sterilization pretreatments (e.g., heat or irradiation) can release charged, leachable, or low-molecular weight fragments, thereby increasing the effective osmotic load locally. Ionically cross-linked biomaterials can exacerbate when they have intentionally added divalent salts (e.g., Ca^2+^, Mg^2+^).

Recent empirical studies have confirmed these effects. Song et al. observed a ∼18 mOsm/kg rise in osmolality over 14 days in chitosan/β-glycerophosphate/collagen hydrogels with adipose-derived stem cells, due to gradual ion redistribution.^102^ Lafuente-Merchan et al. reported 10–15 mOsm/kg increases from sterilized nanocellulose–alginate inks leaching Na^+^/Ca^2+^ in D1 mesenchymal stromal cell culture.^24^ Hypothetically, these polyelectrolyte scaffolds can create Donnan-type ion partitioning at the gel–medium interface, where fixed charges exclude co-ions while attracting counterions, potentially forming local hypertonic microenvironments even when bulk osmolality remains stable.^120^

Polyelectrolyte scaffolds such as alginate, chitosan, or nanocellulose bearing fixed anionic or cationic charges can, in principle, establish Donnan-type ion partitioning at the biomaterial gel–medium interface, where these immobile polymer charges exclude co-ions while attracting counterions from solution, creating asymmetric ion distributions. This could generate local osmotic niches at the scaffold–cell interface, where counterion accumulation could increase total solute concentration without water influx, thereby making the near-surface microenvironment hypertonic relative to the bulk medium. Myoblasts adhering here would therefore experience distinct osmotic conditions that may impair integrin-mediated adhesion, trigger volume-regulatory responses, or delay fusion, potentially mimicking matrix defects rather than direct osmotic stress. Presoaking scaffolds to equilibrate leachable remains is essential to characterize these osmotic niches before cell seeding, ensuring that adhesion and differentiation outcomes reflect true matrix effects.^103,104^ Further work is needed, including direct osmolality profiling at scaffold interfaces using potential microelectrodes, quantitative comparison of myoblast fusion kinetics in preconditioned vs native scaffolds, and systematic testing of counterion effects (Na^+^ vs Ca^2+^) on myotube maturation in alginate/chitosan systems.

Collectively, these interacting drift mechanisms underscore the need to interpret osmolality not as a fixed property but as a system-level outcome, motivating its treatment as a core process parameter. This multiscale perspective ultimately positions osmolality as a unifying variable that links physicochemical behavior, cellular response, and process design across the full spectrum of muscle-cell culture systems.

## 6 Discussion: Osmolality as a Critical Processing Parameter

The analysis presented here reframes osmolality from a descriptive property of culture media to a governing constraint on myogenic cell culture design and operation. Rather than acting independently, physicochemical interactions, cellular metabolism, and process conditions collectively define a dynamic osmotic environment that evolves throughout the culture. As a result, the relevance of osmolality does not arise from its absolute value alone but from when and how systems approach or exceed functional limits relative to the cell state and process context. Although the literature often neglects to explicitly consider osmolality as a controllable parameter across different stages of muscle cell culture and across different scale platforms, this article has made a deliberate effort to synthesize and analyze the available data coherently to build a more bioprocess-relevant framework. Beyond their relevance to cultured meat production, osmotic conditions emerge not only as determinants of cellular performance but also as a structural limit to process stability during extended culture and platform scale-up.

A central insight emerging from this work is that osmolality becomes most critical at the interface between biological sensitivity and process complexity. Muscle-lineage cells inherently operate within a somewhat restricted osmotic tolerance due to their reliance on tightly regulated ion gradients, cytoskeletal organization, and fusion-driven differentiation. These sensitivities are not constant but vary across proliferation, differentiation, and maturation stages, implying that no single “optimal” value is universally applicable. Instead, osmotic requirements are phase-dependent and must be interpreted relative to cellular function rather than viability alone. Seeded, differentiated, and mature muscle cells may therefore respond differently even at the same nominal osmolality, meaning that composition must be optimized experimentally rather than assumed to be theoretically equivalent.

This biological constraint intersects directly with media and process design. In modern serum-free and chemically defined culture media, replacing serum functionality with defined components increases the density and diversity of solutes, effectively compressing the available formulation space. In this context, osmolality defines an upper boundary on compositional freedom: a finite “chemical space” within which nutritional, buffering, and regulatory functions must be balanced. Importantly, this constraint is not solely quantitative. Two different media with equivalent osmolality can impose fundamentally different physicochemical environments depending on the osmolyte identity, interaction strength, and water activity, meaning that formulation equivalence cannot be inferred from total medium osmolality alone.

A second key insight is that osmolality drift is not a single phenomenon but the emergent outcome of multiple coupled mechanisms, each dominant at different scales and under different conditions. Evaporation governs early-stage and small-volume systems; CO_2_–bicarbonate coupling and pH control dominate in controlled environments, while metabolic accumulation and feed strategies drive drift in high-density and long-duration bioreactor cultures. Material interactions introduce an additional layer of complexity by generating localized osmotic heterogeneity that may not be detectable through bulk measurements. These overlapping contributions mean that osmolality trajectories, rather than static values, determine the effective cellular environment (as illustrated in Figure 3).

**Figure 3:**
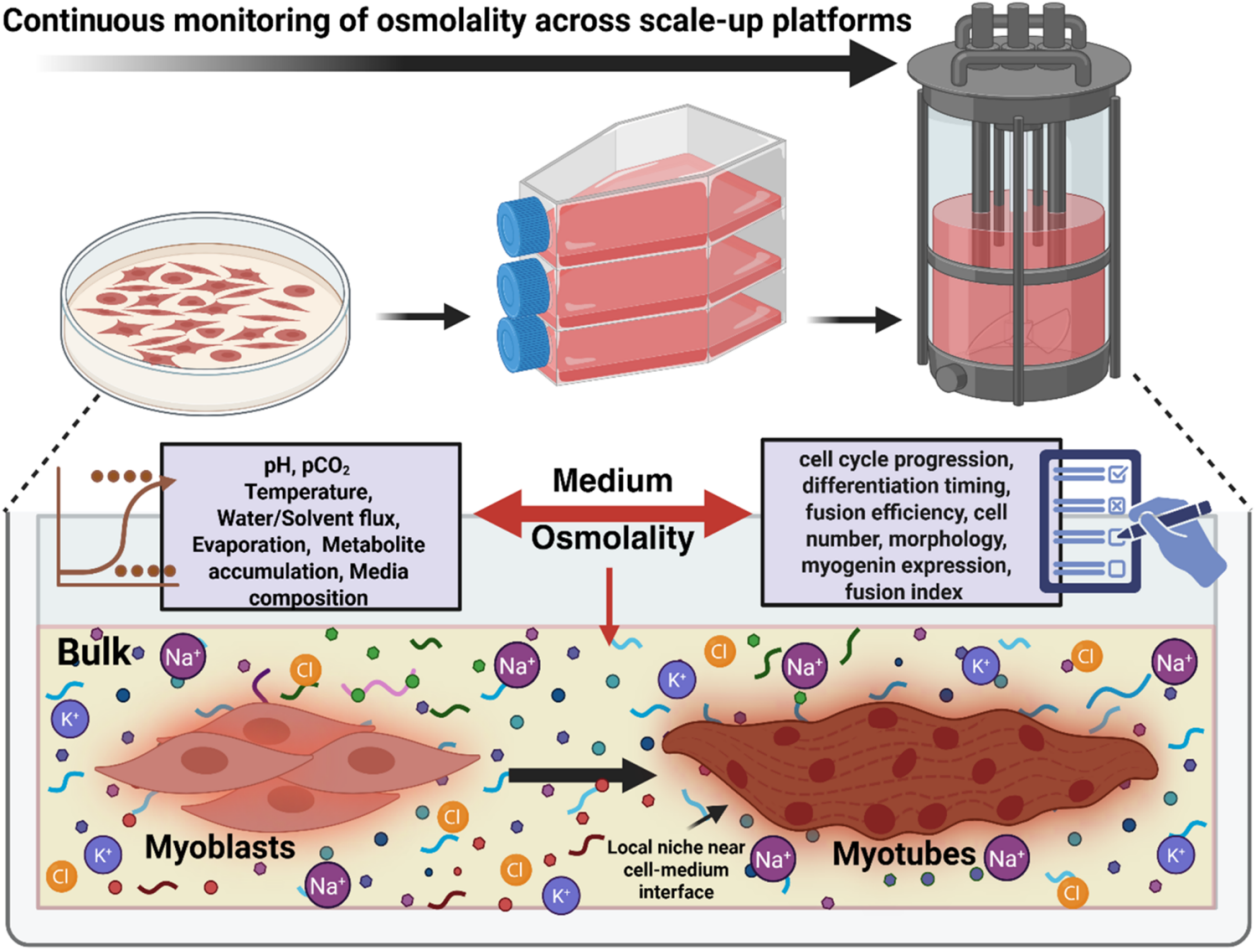
Overview of the complex osmolality landscape governing myogenic culture systems across scale-up workflows. Key biophysical factors influence media osmolality from Petri dish to stirred-tank bioreactor cultures, which directly or indirectly affect various outcomes, from myoblast to myotube proliferation and differentiation. Overall, the figure advocates for consistent, continuous monitoring of medium osmolality across scaling platforms to provide more quantifiable functional information on proliferation indices, differentiation markers, fusion, maturation, morphology metrics, etc. Created with Biorender.com.

From a process perspective, this leads to a fundamental operational setback: osmolality accumulates but is often not readily reduced during the culture. Unlike pH or dissolved oxygen, which can be actively regulated, osmotic increases tend to be path-dependent and irreversible without dilution or medium exchange. Consequently, control strategies must shift from reactive correction to anticipatory design, in which formulation, feeding, gas control, and platform configuration are selected to minimize drift before it occurs. This also means that osmolality should be treated as a core process parameter, similar to pH and dissolved oxygen, with consistent monitoring and defined limits. Reflecting this commercial process, analytics using automated bioanalyzers such as the Cedex Bio- and Cedex Bio HT-derived values for key solutes, including sodium, glucose, and glutamine, rather than directly measuring only the medium’s colligative properties. These measurements capture the major contributors to osmotic balance and are well suited for real-time monitoring of changes during culture. While they do not account for every component in the medium, they provide a consistent and practical approximation of total osmolality that is highly effective for process control.^122^ As such, these systems serve as valuable tools for continuous bioprocess monitoring, enabling tracking of dynamic trends, early detection of drift, and more informed decision-making during culture rather than relying solely on single-point comparisons between media.

In the context of cultivated meat and advanced muscle cell biomanufacturing, these insights become particularly consequential. Efforts to reduce cost, eliminate serum, and extend the culture duration inherently push systems closer to osmotic limits while simultaneously increasing the likelihood of drift and heterogeneity. As a result, osmotic effects that may be negligible in conventional short-term cultures can become dominant constraints under industrial-relevant conditions. Recognizing this shift is critical for achieving reproducibility, scalability, and functional tissue outcomes. Collectively, this work supports a change in the operationalization of osmolality in mammalian cell culture. Rather than being treated as a background parameter, it should be incorporated explicitly into process design, monitoring, and interpretation. In this role, osmolality functions not only as a physical property but also as a unifying descriptor linking medium composition, cellular response, and process dynamics.

Looking forward, integrating osmolality into predictive frameworks represents a logical next step. By coupling osmotic measurements with medium composition, process variables, and phenotypic outputs, it becomes possible to move toward more adaptive and quantitative control strategies. In this context, machine learning approaches can be leveraged to move beyond static medium formulation and toward predictive and adaptive bioprocess design. For example, models trained on medium composition, feeding history, pH, and osmolality profiles, together with culture outcomes, could predict how changes in formulation or operating conditions affect both osmotic behavior and myogenic performance. Such approaches could enable identification of cost-effective formulations within defined osmotic limits, support stage-specific media optimization for proliferation and differentiation, and flag conditions likely to drift outside acceptable ranges during scale-up.

In such frameworks, osmolality is no longer a passive constraint but an actionable design variable that can guide optimization across scales and applications. Ultimately, recognizing when osmolality becomes limiting in experimental and engineering practice will be essential for the development of robust, scalable, and physiologically relevant muscle-cell culture systems.
